# Supplementary material for: Prediction of pathological complete response to neoadjuvant chemotherapy in locally advanced breast cancer by using a deep learning model with 18F-FDG PET/CT
Source: PLoS One. 2023 Sep 14;18(9):e0290543. doi: 10.1371/journal.pone.0290543 (PMC10501592; doi:10.1371/journal.pone.0290543)
Supplement: Attachment — Submitted filename: Response to Reviewers.docx [file pone.0290543.s001.docx]

RESPONSE TO EDİTOR OFFİCE

1. Please ensure that your manuscript meets PLOS ONE's style requirements, including those for file naming. The PLOS ONE style templates can be found at

https://journals.plos.org/plosone/s/file?id=wjVg/PLOSOne_formatting_sample_main_body.pdf and

<https://journals.plos.org/plosone/s/file?id=ba62/PLOSOne_formatting_sample_title_authors_affiliations.pdf>

Formatted As Requested

2. We noticed you have some minor occurrence of overlapping text with the following previous publication(s), which needs to be addressed:

https://www.ejmi.org/pdf/Significance%20of%20NeutrophilLymphocyte%20Ratio%20and%20TrombocyteLymphocyte%20Ratio%20in%20Predicting%20Complete%20Pathological%20Response%20in%20Patients%20with%20Local%20Advanced%20Breast%20Cancer-32029.pdf

<https://journals.plos.org/plosone/article?id=10.1371%2Fjournal.pone.0259622>

The fırst publıcatıon was related but the second publıcatıon was not added to both dıagnosıs and stage related to depth of response ın metastatıc colorectal cancer.

In your revision ensure you cite all your sources (including your own works), and quote or rephrase any duplicated text outside the methods section. Further consideration is dependent on these concerns being addressed.

3. Thank you for stating the following financial disclosure:

no funding

At this time, please address the following queries:

a) Please clarify the sources of funding (financial or material support) for your study. List the grants or organizations that supported your study, including funding received from your institution.

b) State what role the funders took in the study. If the funders had no role in your study, please state: “The funders had no role in study design, data collection and analysis, decision to publish, or preparation of the manuscript.”

c) If any authors received a salary from any of your funders, please state which authors and which funders.

d) If you did not receive any funding for this study, please state: “The authors received no specific funding for this work.”

Please include your amended statements within your cover letter; we will change the online submission form on your behalf.

4. Thank you for stating the following in your Competing Interests section:

NO authors have competing interests

Please complete your Competing Interests on the online submission form to state any Competing Interests. If you have no competing interests, please state "The authors have declared that no competing interests exist.", as detailed online in our guide for authors at http://journals.plos.org/plosone/s/submit-now

This information should be included in your cover letter; we will change the online submission form on your behalf.

5. Please ensure that you include a title page within your main document. You should list all authors and all affiliations as per our author instructions and clearly indicate the corresponding author.

6. Please ensure that you refer to Figure 5 in your text as, if accepted, production will need this reference to link the reader to the figure.

Added to main document.

7. Please include captions for your Supporting Information files at the end of your manuscript, and update any in-text citations to match accordingly. Please see our Supporting Information guidelines for more information: <http://journals.plos.org/plosone/s/supporting-information>.

All requested changes are provıded.

RESPONSE TO REVİEWERS

Reviewer 1

2. The author should add analysis with each index (SUVmax, TLG, MTV, T and N grade,,,,) which have been assessed for the prediction of pCR in previous studies, and compared the result with the performance obtained by the DLA.

Answer: The aim of our study is to predict pCR with only PET/CT hybrid images, so we did not make any additional analysis to the images. As you mentioned, addition of other indexes such as SUVmax, SUVmean, TLG, MTV, T and N grade, HU could make the analysis better. This is the limitation of the study. "Addition of other indexes such as SUVmax, SUVmean, TLG, MTV, T and N grade, HU would make the analysis better." was added in the limitation part of the discussion.

Others. Did the author measure the SUVmax of the primary breast tumor by DICOM format image before converting it to JPEG? It seems confusing from the author's description.

## Answer: The SUVmax values are measured on DICOM format images with Syngo FastView software by drawing VOIs on the tumor. In "2.4. ^18^F- FDG PET/CT Imaging" section of material and method "The SUVmax values of the primary tumor were calculated using the following formula: SUVmax = tracer uptake in ROI / (injected activity/patient weight)." was changed as " The SUVmax values of the primary tumors were calculated on DICOM images with Syngo FastView software by drawing VOIs on the tumors and using the following formula: SUVmax = activity concentration / (injected activity/patient weight)."

Others. Digital Imaging and Communication in Medicine should be DICOM as an abbreviation.

Answer: "DCM" was changed as "DICOM" with the reviewer's comment.

Reviewer 2

2.4. SUVmax values are measured by using ROIs in the data evaluation. This could lead to a missing of the true SUVmax value. However, implementing image morphological markers such as tumor size, SUVmax, HU might improve the deep learning process.

Answer: SUVmax values are measured by drawing VOIs. The aim of our study is to predict pCR with only PET/CT hybrid images, so we did not make any additional analysis to the images. As you mentioned, addition of other indexes such as SUVmax, SUVmean, TLG, MTV, T and N grade, HU could make the analysis better. This is the limitation of the study.	In "2.4. ^18^F- FDG PET/CT Imaging" section of material and method "The SUVmax values of the primary tumor were calculated using the following formula: SUVmax = tracer uptake in ROI / (injected activity/patient weight)." was changed as " The SUVmax values of the primary tumors were calculated on DICOM images with Syngo FastView software by drawing VOIs on the tumors and using the following formula: SUVmax = activity concentration / (injected activity/patient weight)."

"Addition of other indexes such as SUVmax, SUVmean, TLG, MTV, T and N grade, HU would make the analysis better." was added in the limitation part of the discussion.

-The major limitation is the reduced number of participants included in this study. Does every patient receive the same number of PET/CT examinations to avoid bias?

Answer: In "2.4. ^18^F- FDG PET/CT Imaging" section of material and method "All patients had one PET/CT imaging in the staging of breast cancer before NAC." and "Thirty one PET/CT imagings of 31 patients were used for deep learning process." sentences were added.
